# Lifestyle Patterns of Children Experiencing Homelessness: Family Socio-Ecological Correlates and Links with Physical and Mental Health

**DOI:** 10.3390/ijerph192316276

**Published:** 2022-12-05

**Authors:** Alexandra Descarpentrie, Mégane Estevez, Gilles Brabant, Stéphanie Vandentorren, Sandrine Lioret

**Affiliations:** 1 Université Paris Cité, Inserm, INRAE, Centre for Research in Epidemiology and StatisticS (CRESS), F-75004 Paris, France; 2 Université de Bordeaux, Inserm, UMR1219, PHARes team, F-33000 Bordeaux, France; 3 Santé Publique France, French National Public Health Agency, F-94415 Saint-Maurice, France; 4 Institut Convergences Migration/CNRS, F-93322 Aubervilliers, France

**Keywords:** lifestyle patterns, homelessness, children, family, socio-ecological, health

## Abstract

Diet, screen time, physical activity, and sleep combine into lifestyle patterns with synergistic effects on health. This study aimed to identify lifestyle patterns in children without housing and assess their associations with physical and mental health and family socio-ecological factors. In the 2013 ENFAMS cross-sectional survey (children aged 6–12 experiencing homelessness, Greater Paris area, n = 235), parents reported socio-ecological factors, children’s behaviours, and mental health (the latter was also child-reported). Nurses measured children’s haemoglobin concentrations and body mass index. Principal component analysis was used to derive sex-specific lifestyle patterns. Hierarchical linear regressions and “outcome-wide” analyses assessed, respectively, these patterns’ relations to health and family socio-ecological factors. A rather healthy lifestyle pattern—similarly characterized by diverse diet and high sleep time—was identified, with slight differences by sex. Scores for this pattern were higher for children in food-secure or higher-income households, whose parents were proficient in French, who slept longer, or who received more social support compared to their counterparts, with some nuances by sex. Higher scores for this pattern were associated with higher prosocial behaviour scores (girls) and lower anxiety and hyperactivity–inattention symptoms scores (boys), but not with physical health. For this underserved and understudied population, the results highlight the importance of family socio-ecological factors in shaping the lifestyles and mental health of children.

## 1. Introduction

Development occurs throughout the lifespan, but the earliest phases of maturation in the first years of life are particularly critical. A growing body of literature describes the lasting negative consequences for lifelong health of exposure to adverse events (e.g., experiencing homelessness, abuse, or witnessing violence) during childhood [1]. Worrisomely, families with children are the fastest growing population undergoing homelessness in many European countries [2], including France. Previous studies have demonstrated that children from these families are disproportionately burdened by poor physical and mental health relative to their housed counterparts [3,4]. 

Homelessness is an obvious obstacle to the preservation of the essence of family life—a stable and secure place for nurturing children. Parents without homes face intersecting structural barriers—such as poverty, food insecurity, and lack of access to care—that undercut their ability to protect and guide their children. This engenders feelings of anxiety, guilt, and shame [5]. This psychosocial vulnerability may further exacerbate suboptimal parenting practices, including those related to children’s energy balance-related behaviours (EBRBs); i.e., diet, screen time, physical activity, and sleep [6]. Nonetheless, EBRBs are potentially modifiable behaviours that, when optimal, may foster children’s health [7,8].

Studies documenting EBRBs in children experiencing homelessness and their correlates and links with health are sparse [9,10]. There is no evidence about how these behaviours, when combined into lifestyle patterns, exert their effects, which may be synergistic, on children’s health in these deprived living conditions [11]. General population studies have highlighted three types of child lifestyle patterns: unhealthy, healthy, or mixed, with some nuances by sex [12]. The unhealthy lifestyle pattern, often characterized by high consumption of discretionary food and sugar-sweetened beverages (SSBs) and high screen time, is the most consistently identified unhealthy pattern worldwide. In high-income countries, adherence to it has been shown to be inversely associated with parental socio-economic position (SEP) and positively with obesity risk [12]. Evidence about other physical or mental health dimensions or family socio-ecological factors is, however, limited.

This study aimed to gain a greater understanding of lifestyle patterns, their family socio-ecological correlates, and their relations to physical and mental health among girls and boys experiencing homelessness. 

## 2. Methods

### 2.1. Study Design and Participants

The cross-sectional *Enfants et familles sans logement* (ENFAMS) survey [13] was conducted by the *Observatoire du Samu social de Paris* from January to May 2013 to describe the social and health characteristics of homeless families in the Greater Paris area (approximately 12 million inhabitants). Specific focuses were placed on mental health, nutrition (especially food insecurity and anaemia), child development and access to health care for parents and children. Of 251 shelters randomly selected, 14 were outside the scope of the survey (8 did not house families, 5 closed early in 2013, and 1 was a duplicate) and 193 agreed to participate (82% participation rate). They comprised emergency centres, long-term rehabilitation centres, social hostels, and centres for asylum seekers; of the 1238 randomly selected families, 801 agreed to participate in the survey (65% participation). One child younger than 13 years (and a parent—the mother when present) was randomly selected to participate. Families were offered vouchers for their participation. Written consent was obtained from each family and each parent of the children who participated, and it was made clear, orally and in writing, that refusal to participate in the survey had no impact on the care of families or on accommodation. Here, we studied families with at least one child aged 6–12 years (n = 235), as EBRBs of interests have not been ascertained in children aged <6 years. 

### 2.2. Measures and Questionnaires 

Children’s EBRBs and family socio-ecological data were collected in face-to-face interviews with the parent (97% mothers). As most of parents were migrants, trained interviewers conducted these surveys in 17 languages [13]. 

#### 2.2.1. Children’s EBRBs

*Diet*. Information regarding the child’s usual dietary intake was collected with a 15 item semi-quantitative food frequency questionnaire covering part of the diet. For each food item, four possible responses were converted into weekly frequencies (“Every day” was coded as 7 times/week, “Several times per week” as 3.5, “Less often” as 1, and “Never” as 0). When relevant, similarities in food type and the context of consumption were used to define larger food groups and to convert the summed frequencies. This process produced the following eight groups: dairy products, fish, fruit (fresh and stewed), vegetables (raw and cooked), rice and pasta, bread, SSBs (sodas and fruit juice), and French fries. 

*Screen time*. The time the child spent watching TV or DVDs or playing computer/video games was collected for typical school (Monday, Tuesday, Thursday, Friday) and non-school (Wednesday, Saturday, Sunday) days from the week before the interview. The four response options were converted into daily equivalents: “>2 h/day” was coded as 2.5; “1 to 2 h/day” as 1.5; “<1 h/day” as 0.5; and “Never” as 0). Screen time (hours/day) was computed as [(school day × 4) + (non-school days × 3)]/7.

*Outdoor play*. Outdoor play was reported as the number of days the child played outdoors outside school hours (including informal unsupervised activities, such as playing in a playground, in front of the shelter, rollerblading, etc.) on school and non-school days from the week before the interview. Accordingly, after summing these quantities, frequency of outdoor play was expressed in days/week.

*Sleep*. Usual night sleep time was computed from the typical bedtime and waking time in hours/day.

#### 2.2.2. Family Socio-Ecological Factors

**Figure 1:**
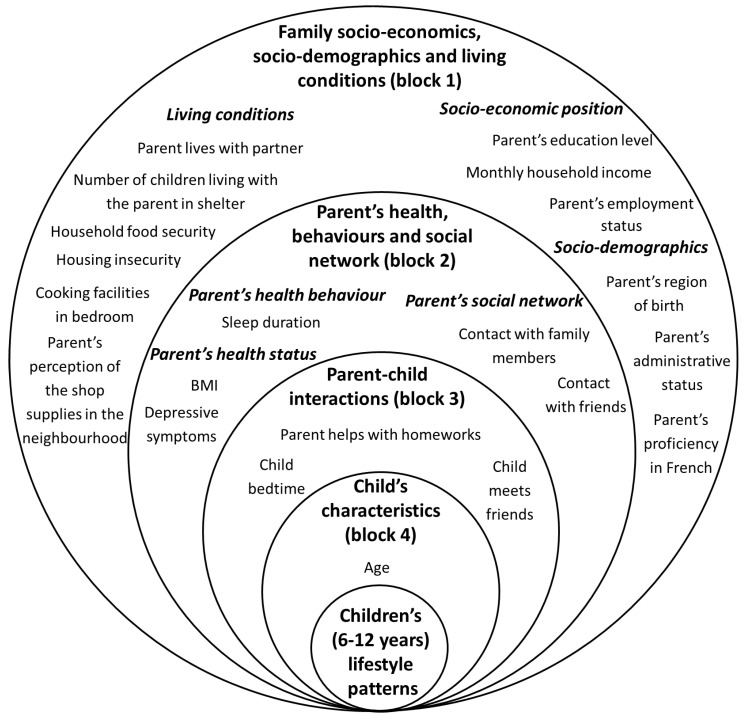
Socio-ecological model for correlates of lifestyle patterns among homeless children (6–12 years). Adapted from Descarpentrie et al., 2021, and Frenoy et al., 2021 [14,15].

We adapted conceptual frameworks developed both in homeless settings and general populations to serve as an overarching guide for understanding how family socio-ecological factors can affect children’s lifestyle patterns [14,15]. The resulting model, presented in Figure 1**,** is divided into four levels: (1) socio-economic, sociodemographic, and living conditions; (2) parents’ health, behaviours, and social networks; (3) parent–child interactions; and (4) children’s characteristics. Supplementary Material S1 provides further details.

#### 2.2.3. Children’s Physical Health

*Haemoglobin concentration*. Nurses immediately measured haemoglobin concentration from a capillary blood sample using a portable haemoglobinometer (HemoCue Hb201+ System, Angelholm, Sweden) [16].

*Body mass index (BMI)*. Nurses measured children’s weight using a calibrated SECA balance scale and length or height with a sliding foot scale or a wall-mounted stadiometer, with precision of 0.1 kg and 0.1 cm, respectively. Height and weight were measured while participants were dressed in light indoor clothing without footwear. BMI z-scores, adjusted for age and sex, were calculated (WHO references) [17]. 

#### 2.2.4. Children’s Mental Health

*Child-reported.* The Dominic Interactive (DI) assessment is a self-administered computerized questionnaire relevant for children aged between 6 and 13 years old [18]. It assesses the emotional and behavioural symptoms of seven childhood mental disorders according to the Diagnostic and Statistical Manual—Revision 4 (DSM-IV) using 91 items and scores for specific phobias, separation anxiety disorder, generalized anxiety disorder, major depressive disorder (internalizing problems), attention deficit/hyperactivity disorder, conduct disorder, and oppositional defiant disorder (externalizing problems). Questions about children’s strengths and competencies are also included. 

*Parent-reported.* The Strength and Difficulties Questionnaire (SDQ) is a screening questionnaire for children aged 4–16 years comprising 25 items distributed across five subscales (each with five items): emotional symptoms and peer relationship problems (internalizing problems); conduct and hyperactivity–inattention problems (externalizing problems); and prosocial behaviours [19]. 

#### 2.2.5. Other Variables

For the lifestyle patterns–health analyses, we additionally accounted for the following correlates [20]: parent age (continuous), parent anaemia (yes, no), parent exposure to domestic violence in the preceding 12 months (yes, no), the child’s reports of being bullied at school (yes, no), whether the child disliked the family’s accommodation (yes, no), whether the child had a health problem that requires specific care (yes, no), and child birthweight (continuous).

#### 2.2.6. Statistical Analysis

*Descriptive statistics.* We described the families’ socio-ecological factors and the children’s health outcomes; after multiple imputation, proportions and means were weighted inversely to each participant’s inclusion probability. 

*Lifestyle patterns*. We synthesized the 11 EBRBs of interest into lifestyle patterns using principal component analysis (PCA) separately for girls and boys [12]. Missing data (14% in girls, 25% in boys) were imputed with the regularized iterative PCA method [21]. Examination of eigenvalues, scree plots, and parallel analysis results, along with the interpretability of derived patterns, determined the utility of components in further analyses [22]. Items with a loading with an absolute value >0.20 were considered to make a reasonable contribution to the principal component, which was interpreted and labelled accordingly. Each child had a score for each lifestyle pattern: a higher score represented higher “adherence” (either intentional or unintentional).

*Family socio-ecological correlates*. As planned with our socio-ecological model (Figure 1), we ran hierarchical linear regressions to assess the relations between family socio-ecological factors and the children’s lifestyle patterns [23]. First, unadjusted analyses were conducted to examine the associations between factors and lifestyle patterns in each level. Variables in the distal level (level 1) that reached a *p* value <0.20 in the unadjusted analyses were included in the adjusted regression (model 1). Subsequently, variables in the next level (level 2) with *p* < 0.20 in the unadjusted analyses were subjected to the adjusted regression together with the distal variables (level 1) retained in the previous step (model 1), and so on. This approach minimized the likelihood that intermediate variables would affect the relations between the distal variables and the lifestyle patterns.

*Links with health*. Linear regression models assessed the associations between lifestyle patterns and children’s standardized health outcomes. We drew on the outcome-wide approach [24] to run various analyses adjusted for a common set of household, parent, and child factors known to be linked to child health (details on this set are presented in the notes of the related Table – see *Results* Section 3.3 *Links with Health)*.

*Missing data*. To prevent information loss and selection biases, missing values for health outcomes, family socio-ecological factors, and other potential confounders were multiply imputed (Supplemental Material S2).

*Sensitivity analyses*. We first carried out PCA for children with complete data for all EBRBs (girls, n = 98; boys, n = 91). Next, to test whether the input variables violated the normal distribution hypotheses, we performed PCA on the polychoric correlation matrix derived after considering the (initial) categorized EBRBs [25]. To optimize the clinical interpretation, we further dichotomized health outcomes according to well-defined cut-offs and replicated our main analysis. Finally, to evaluate the robustness of our effect estimates to unmeasured confounding, we calculated *E-values* for each lifestyle pattern–outcome association [24]. 

All statistical analyses were stratified by sex and performed with R software, which took into account the multiply imputed data and the sampling design.

## 3. Results

Fewer than 2% of caregivers were born in France and about 70% of them were living with their partner. Families’ average monthly income was EUR 325.6/consumption unit (CU) (95% confidence interval (CI): 271–380.3). Living conditions varied: 10.5% (95% CI: 6.0–15.5) of the families were food-secure, and 22.0% (95% CI: 14.9–29.0) had moved more than twice in the past year. Children’s average age was 8.8 years (95% CI: 8.4–9.1). Table 1 further details family socio-ecological factors and children’s health outcomes by sex.

### 3.1. Lifestyle Patterns 

A common and rather healthy lifestyle pattern identified in both sexes explained around 20% of the total variance (Table 2). It was highly correlated with the consumption of almost all the food groups considered (fish, dairy products, fruit, vegetables, rice/pasta, SSBs, and French fries) and sleep time. This pattern was labelled “Diverse diet, sleep” in girls and “Diverse diet, sleep, outdoor play” in boys, with the latter being further characterized by plentiful outdoor play time. A mixed pattern (explaining around 11% of the total variance) was additionally identified in boys and defined by high consumption of SSBs and bread and plentiful outdoor play and sleep time but low rice/pasta and fish consumption; it was termed “Unbalanced diet, outdoor play, sleep”. 

### 3.2. Family Socio-Ecological Correlates

Children reared in families earning more than EUR 28/CU/month scored higher for the rather healthy lifestyle pattern than those from families earning less (β coefficient 1.03 (95% CI: 0.27–1.08) in girls; β 0.77 (95% CI: 0.18–1.35) in boys) (Table 3). Girls whose parents were proficient in French, whose parents had regularized administrative status (in possession of refugee status or a residence permit application receipt or without a valid residence permit/document), or who had more hours of sleep had higher scores for this pattern than those without each of these factors (respectively, β 0.64 (95% CI: 0.08–1.20); β 1.25 (95% CI: 0.76–1.74); β 0.17 (95% CI: 0.08–0.25)). Boys living with an employed parent (β 0.62 (95% CI: 0.02–1.22)), in a food-secure household (β 1.02 (95% CI: 0.23–1.81)), or with a parent invited out by friends more than once in the past year (β 0.61 (95% CI: 0.13–1.10)) also had higher scores for this lifestyle pattern compared to their counterparts. Mixed pattern scores were lower for boys who had a late bedtime and higher for those who interacted with friends compared to those who did not (β −0.31 (95% CI: −0.58–−0.04) and β 0.67 (95% CI: 0.18–1.17), respectively).

### 3.3. Links with Health 

Neither BMI z-scores nor haemoglobin concentrations were related to any of the lifestyle patterns (Table 4). However, results revealed that the higher the score for the rather healthy pattern was for girls, the fewer their peer relationship problems (β −0.24 (95% CI: −0.40–−0.09)) and the higher their score was for prosocial behaviours (β 0.31 (95% CI: 0.17–0.45)). In boys, scores for the rather healthy pattern were inversely related to parent-reported hyperactivity–inattention symptoms (β −0.20 (95% CI: −0.34– −0.06)), as well as specific phobias and both separation and generalized anxiety symptoms (β −0.20 (95% CI: −0.39–−0.01), β −0.22 (95% CI: −0.37–−0.06), β −0.21 (95% CI: −0.39–−0.04)). Furthermore, boys who scored higher for the mixed pattern had significantly lower emotional problem scores (β −0.32 (95% CI: −0.50–−0.14)). 

### 3.4. Sensitivity Analyses

First, PCA on complete cases and the polychoric correlation matrix generated similar lifestyle patterns (Supplementary Tables S3 and S4). Second, dichotomization of outcomes yielded consistent findings overall (Supplementary Table S5), despite a loss of statistical power. Finally, *E-values* (mostly ranging between estimates of 1.20 and 2.01) suggested that associations between lifestyle patterns and the various outcomes were at least moderately robust to potential unmeasured confounders (Supplementary Table S6). 

## 4. Discussion

Our results indicate that the covariance of EBRBs in children experiencing homelessness is primarily explained by the healthiness of their lifestyle patterns: those scoring the highest seemed to have a more diverse diet and sleep for longer times (with more frequent outdoor play for boys as well) and vice versa for those with the lowest scores. A higher score for this pattern was associated with more favourable socio-ecological family factors but also with better socio-emotional and behavioural outcomes.

### 4.1. Lifestyle Patterns

Although classified as rather healthy or mixed, the lifestyle patterns identified in this study are partly atypical in their characterization, with no strict equivalent commonly identified in the general paediatric population. Nonetheless, the sex disparity for outdoor play in this homeless population was consistent with the one observed in the general population; physical activity is only an important contributor to lifestyle patterns for boys [12]. Unlike findings in the general population, screen time characterized none of the identified patterns. This may be explained in part by the fact that housing instability is not compatible with settled habits for TV-based viewing (devices are not available in all shelters). The definition of the rather healthy lifestyle pattern by high consumption of both nutrient-dense and energy-dense foods could also reflect the duality between the specificities related to the socio-cultural background of migrant families (healthy traditional foods) and suboptimal food environments (satiating junk foods at low prices) [26]. 

### 4.2. Family Socio-Ecological Correlates

Proficiency in French can be interpreted as an indicator of acculturation, and its positive association with the rather healthy lifestyle pattern in girls may suggest that the children’s traditional eating habits (usually plant-based) were gradually switching towards Westernized diets [27]. Alternatively, parents without housing, with limited French proficiency, and confronted by a Western obesogenic environment may face barriers to benefiting from information and to attaining opportunities for optimal EBRB options for their family [28]. Having access to and understanding health information is not sufficient: it is equally important to be able to use this information properly to enhance health. Employed parents are likely to be more self-confident and receive more social support compared to those who are unemployed; alternatively, employment may reflect easier access to tangible opportunities (flexible workplace arrangements, out-of-school childcare) [29]. 

Regardless of financial constraints, household food security may denote favourable distal circumstances; i.e., neighbourhoods with more diverse food retailers or optimal food environments within shelters. Indeed, only optimal food environments are able to support parents’ ability to acquire sufficient, safe, diverse, and nutritious foods through socially acceptable means [30], as reflected by the results among boys. Conversely, food insecurity is likely a stressful life experience: children in food-insecure households may not only perceive their family’s limited food resources but may also be psychologically affected by this restriction [31] in ways that impair their eating and sleep habits [6]. 

Social support may serve to alleviate parenting stress in social adversity: invitations by friends can be presumed to provide tangible support to parents if their friends also have children with whom theirs can play. In neighbourhoods with high concentrations of families facing social vulnerabilities, the impoverishment within social networks tends to ripple outwards, and local institutions, such as schools, parks, community centres, and libraries, gradually deteriorate [32]. In more affluent communities, these institutions offer opportunities for families in their child-rearing tasks [32]. Moreover, social support from caring friends with nurturing experience in parenting roles is likely to also provide valuable insights into optimal parenting skills [33]. 

Modelling describes the process of adopting behaviours through watching and imitating others, such as parents [34]. As suggested by the results for girls and further corroborated by those for the general population [14], modelling may additionally constitute a relevant mechanism linking parents’ sleep time to that of their children. Potentially, non-sleep-deprived caregivers may also have higher parenting self-efficacy levels that favour optimal EBRB-related rule setting. 

Caregivers’ rules may help children build solid habits: parents who stick to a set bedtime routine appear to have children who get enough sleep, as suggested by the association observed between boys’ early bedtime and higher scores for the mixed pattern [35]. On the other hand, these results may also reflect environmental factors, such as shelter rules, sharing a bed with other family members, or a noise-polluted environment—all of which impair the sleep hygiene of both parents and children. 

### 4.3. Lifestyle Patterns and Health

Regardless of the likely influence of upstream (living) conditions, the rather healthy lifestyle pattern appears to promote more prosocial behaviours and prevent both internalizing and externalizing symptoms downstream. The functioning of the prefrontal cortex, closely linked to executive function, is highly susceptible to sleep deprivation, as well as to suboptimal physical activity and diet [36,37]. Moreover, executive function has been related to both internalizing and externalizing problems [38] and, hence, potentially to children’s ability to prevent socio-emotional difficulties. In both sexes, associations were congruent for internalizing problem scales but not for relations with prosocial behaviours and hyperactivity–inattention symptoms. As they develop language and theory-of-mind skills earlier than boys do [39], girls are likely to be more skilled at decoding others’ emotions and understanding others’ thoughts, while boys are more likely than girls to exhibit motor-related hyperactivity [40]. Relatively low parent–child consistency was observed for lifestyle pattern associations with internalizing and externalizing symptoms, which is unsurprising in view of the documented lack of between-informant agreement [41]. Nevertheless, the two lifestyle patterns identified in boys—both characterized by plentiful sleep and outdoor play time—were associated with child- and parent-reported internalizing problem scales. This similarity suggests some consistency between the two assessments, perhaps because the beneficial roles of these two EBRBs in these associations outweigh the roles of the other factors. Lastly, the absence of an association in the current study between lifestyle patterns and the child’s physical health (BMI and haemoglobin concentration) may be partly explained by the both optimal and suboptimal EBRBs combining into the lifestyle patterns, along with the study’s cross-sectional design. Longitudinal research, tough and challenging to conduct within such settings, given the residential instability of families experiencing homelessness, may provide further insights about links between lifestyle patterns and long-term health.

### 4.4. Public Health Implications

Whereas the mixed lifestyle pattern–emotional health association for boys requires further examination, the current findings highlight that the promotion of a healthy—or even somewhat healthy—combination of EBRBs while adopting a family-centred approach is promising for the facilitation of better health in children experiencing homelessness. As the continuum of risk hypothesis suggests [42], however, in such deprived living conditions, (social) health correlates share commonalities with the (social) structural determinants of health inequalities in the general population but differ in their degree in the presence of multiple intersecting (specific) determinants. Poor housing quality, the high cost of housing, housing instability, and few neighbourhood opportunities can lead to a cumulative burden by interacting, often synergistically, with one another and with other structurally rooted inequalities and produce and reify health disparities. Simply obtaining adequate and stable housing is not, however, sufficient. Parents who have had lives with multiple stressors, such as migration to escape from violence or distancing from social norms, may need many life skills, strategies, and resources to parent successfully [33]. One critical strategy may involve empowering caregivers with essential resources; namely, skills, knowledge, financing, and non-stigmatizing social support. 

### 4.5. Limitations and Strengths

This study is primarily limited by its cross-sectional design, which precludes any causal interpretation. Likewise, the relatively small sample size may have limited its statistical power. Using an outcome-wide lens that has greater potential to report null effects [24], this research provides valuable evidence about links between lifestyle patterns and various health outcomes among this understudied population. Additionally, this work rigorously considered a wide array of covariates, which helped to reduce potential confounding and selection bias, though the possibility of broader (outcome-specific) unmeasured confounding cannot be entirely ruled out. Other key strengths of our study were the use of validated child psychological questionnaires (the SDQ and DI assessment) and objective measures of physical health that help to mitigate reporter bias, as well as the consistency of the highlighted lifestyle pattern–mental health associations with those underlined in the general population [43]. This research may seem relatively dated given the rapid changes that have occurred since the ENFAMS study [2]. This vulnerable population is, however, hard to survey. Thus, the ENFAMS study remains unique in France, considering its high-quality methodology (sampling design, participation rate, interpreters for a wide range of languages), and still relevant, as the number of families living without housing continues to grow.

## 5. Conclusions

Among families experiencing homelessness, we highlighted positive associations between optimal structural factors at the family level (food security and income), as well as more proximal ones (modelling, social support), and a rather healthy lifestyle pattern. This pattern was also related to better mental health in children. These findings should inform community and policy-level interventions to improve the mental health and well-being of children living in extremely vulnerable conditions.

## Figures and Tables

**Table 1:** Family socio-ecological factors and health characteristics (weighted mean or percentage [95% CI]).^1^ The ENFAMS survey.

	Girls (*n* = 114)	Boys (*n* = 121)
**Socio-economic, sociodemographic, and living conditions**		
***Socio-economic position***		
Parent’s education level (≥high school diploma)	29.9 [19.9–39.9]	33.3 [22.5–44.2]
Parent’s employment status (yes)	29.2 [19.9–38.5]	26.9 [16.7–37.1]
Household monthly income (>EUR 28/CU—T2 and T3)	76.0 [65.3–86.7]	78.6 [69.4–87.8]
***Socio-demographics***		
Parent’s proficiency in French (yes)	34.9 [22.5–47.2]	30.4 [19.7–41.1]
Parent’s region of birth (Sub-Saharan Africa)	34.7 [22.5–46.9]	31.3 [20.9–41.6]
Parent’s administrative status (regularized)	56.0 [45.4–66.6]	44.8 [33.5–56.1]
***Living conditions***		
Parent lives with partner (yes)	65.5 [55.0–76.1]	69.5 [54.9–84.0]
Number of children living with the parent in shelter (>1)	87.4 [81.3–93.6]	83.4 [76.8–90.0]
Household food security (yes)	9.5 [2.8–16.2]	12.0 [4.5–19.5]
Housing insecurity (>2 moves in the last year)	19.6 [10.3–29.0]	24.3 [14.5–34.1]
Cooking facilities in bedroom (yes)	33.8 [21.6–46.1]	28.7 [20.5–36.8]
Parent’s perception of neighbourhood shop supplies (enough)	76.7 [68.0–85.5]	79.0 [70.8–87.1]
**Parent’s health, behaviours, and social network**		
***Parent health***		
Depression symptoms (yes)	36.7 [25.1–48.3]	21.3 [11.8–30.9]
BMI (kg/m^2^)	29.97 [29.07–30.88]	29.63 [28.26–31.01]
***Parent’s behaviour***		
Sleep time (hours)	6.49 [6.06–6.92]	6.55 [6.06–7.04]
***Parent’s social network***		
Contact with all family members (PCA score)	−0.13 [−0.37–0.09]	0.23 [−0.04–0.50]
Contact with friends (≥1 in the last 12 months)	58.0 [45.8–70.3]	53.7 [42.8–64.6]
**Parent–child interactions**		
Child’s bedtime (hours)	21.29 [21.13–21.45]	21.29 [21.12–21.47]
Parent helps with homework (>30 min/day)	31.6 [20.2–42.9]	38.3 [26.6–50.0]
Child meets friends (yes)	75.0 [62.6–87.4]	73.1 [63.1–83.2]
**Child’s characteristics**		
Child’s age (years)	9.04 [8.61–9.46]	8.52 [8.19–8.85]
**Child’s health outcomes**		
***Physical health***		
BMI z-score (WHO references)	0.83 [0.59–1.08]	0.63 [0.34–0.92]
Haemoglobin concentration (g/dL—range: 8–15)	11.65 [11.28–12.02]	11.69 [11.47–11.90]
***Mental health***		
***Child-reported (DI scores)***		
*Internalizing*		
Specific phobia symptoms (range: 0–9)	2.87 [2.49–3.25]	2.36 [1.61–3.11]
Separation anxiety symptoms (range: 0–8)	3.97 [3.53–4.41]	3.31 [2.76–3.86]
Generalized anxiety symptoms (range: 0–15)	7.24 [6.11–8.36]	6.48 [5.37–7.58]
Depression/dysthymia symptoms (range: 0–20)	6.65 [5.68–7.62]	6.50 [5.12–7.88]
*Externalizing*		
Opposition symptoms (range: 0–9)	2.50 [2.09–2.90]	2.85 [2.11–3.58]
Conduct problems (range: 0–14)	1.40 [0.91–1.89]	2.58 [1.56–3.59]
Hyperactivity–inattention symptoms (range: 0–19)	5.57 [4.80–6.35]	5.92 [4.43–7.41]
*Well-being*		
Strength and competencies (range: 0–10)	8.64 [8.28–9.00]	8.34 [7.68–9.00]
***Parent-reported (SDQ scores)***		
*Internalizing*		
Emotion symptoms (range: 0–10)	3.54 [2.98–4.09]	3.54 [2.92–4.15]
Peer relationship problems (range: 0–10)	2.19 [1.72–2.66]	1.98 [1.60–2.37]
*Externalizing*		
Conduct problems (range: 0–10)	1.73 [1.29–2.19]	2.17 [1.77–2.57]
Hyperactivity–inattention symptoms (range: 0–10)	3.39 [2.83–3.94]	4.10 [3.54–4.65]
*Well-being*		
Prosocial behaviours (range: 0–10)	8.90 [8.53–9.27]	8.35 [7.80–8.90]

BMI: body mass index; CI: confidence interval; CU: consumption unit; DI: Dominic Interactive, SDQ: Strength and Difficulties Questionnaire; T: tertile; WHO: World Health Organization. ^1^ When variables are binary, only the distribution for one modality is displayed.

**Table 2:** Distribution of EBRBs and principal component factor loadings for lifestyle patterns in girls and boys aged 6–12 years. The ENFAMS survey.

	Girls (*n* = 114)		Boys (*n* = 121)		
	Mean (SD)	LP1	Mean (SD)	LP1	LP2
Yogurts ^1^	4.71 (2.39)	**0.59**	4.12 (2.43)	**0.49**	0.01
Fish ^1^	2.03 (1.57)	**0.53**	2.16 (1.63)	**0.46**	**−0.50**
Fruit ^1^	6.90 (3.67)	**0.60**	6.07 (3.52)	**0.74**	0.00
Vegetables ^1^	5.52 (3.09)	**0.49**	5.12 (3.21)	**0.68**	0.00
Rice/pasta ^1^	4.78 (2.16)	**0.28**	4.75 (1.92)	**0.50**	**−0.33**
Bread ^1^	5.70 (2.24)	**0.30**	5.79 (2.05)	0.00	**0.65**
SSBs ^1^	6.41 (3.85)	**0.66**	6.68 (3.78)	**0.43**	**0.31**
French fries ^1^	2.41 (1.51)	**0.40**	2.38 (1.97)	**0.50**	0.07
Screen time ^2^	1.88 (0.88)	−0.01	1.96 (0.87)	−0.13	0.10
Outdoor play ^3^	2.18 (2.20)	−0.06	2.43 (2.28)	**0.24**	**0.53**
Sleep ^2^	9.76 (0.95)	**0.34**	9.55 (1.07)	**0.21**	**0.44**
Variance explained (%)		19.6		21.0	12.6
Label		Diverse diet, sleep		Diverse diet, outdoor play, sleep	Unbalanced diet, outdoor play, sleep

SSBs: sugar-sweetened beverages; LP: lifestyle pattern; SD: standard deviation. ^1^ Times/week. ^2^ Hours/day. ^3^ Days/week. In bold, factor loadings >0.20 or <−0.20.

**Table 3:** Associations (β [95% CI]) between family socio-ecological factors and lifestyle patterns in girls and boys: unadjusted and adjusted models. The ENFAMS survey.

	Girls (*n* = 114)		Boys (*n* = 121)			
	Diverse Diet, Sleep		Diverse Diet, Outdoor Play, Sleep		Unbalanced Diet, Outdoor Play, Sleep	
	Unadjusted	Adjusted ^1,2,3,4^	Unadjusted	Adjusted ^1,2,3,4^	Unadjusted	Adjusted ^1,2,3,4^
**Socio-economic, sociodemographic, and living conditions (model 1) ^1^**						
***Socio-economic position***						
Parent’s education level (≥ vs. < high school diploma)	0.00 [−0.67–0.66]		0.00 [−0.71–0.72]		0.32 [−0.22–0.85]	
Parent’s employment status (yes vs. no)	1.05 [−0.09–2.19]	0.14 [−0.74–1.03]	0.91 [0.42–1.41]	0.62 [0.02–1.22]	−0.55 [−1.06– −0.04]	−0.45 [−1.01–0.11]
Household monthly income (> vs. ≤ EUR 28/CU—T1)	1.92 [1.18–2.67]	1.03 [0.27–1.80]	1.16 [0.41–1.92]	0.77 [0.18–1.35]	0.28 [−0.15–0.72]	
***Socio-demographics***						
Parent’s proficiency in French (yes vs. no)	1.12 [0.47–1.17]	0.64 [0.08–1.20]	0.50 [−0.18– 1.18]	0.18 [−0.48–0.85]	0.15 [−0.44–0.75]	
Parent’s region of birth (Sub-Saharan Africa vs. other countries)	1.03 [0.43– 1.64]	0.20 [−0.31– 0.71]	0.04 [0.52– −0.59]		−0.51 [−0.98– −0.03]	−0.28 [−0.79–0.23]
Parent administrative status (regularized vs. not)	1.72 [1.13–2.31]	1.25 [0.76–1.74]	0.68 [0.08–1.29]	0.27 [−0.32–0.85]	0.04 [−0.48–0.56]	
***Living conditions***						
Parent lives with partner (yes vs. no)	0.31 [−0.43–1.05]		−0.06 [−0.68–0.56]		0.46 [−0.09–1.01]	0.25 [−0.40–0.90]
Number of children living with the parent in shelter (> vs. =1)	0.38 [−0.21–0.96]		−0.08 [−0.72–0.55]		−0.17 [−0.70–0.35]	
Household food security (yes vs. no)	0.86 [0.14–1.59]	0.15 [−0.63–0.95]	1.09 [0.49–1.69]	1.02 [0.23–1.81]	−0.22 [−0.61–0.17]	
Housing insecurity (> vs. ≤2 moves in the last year)	−0.75 [−1.51–0.00]	0.22 [−0.45–0.88]	−0.26 [−0.81–0.29]		−0.03 [−0.57–0.5]	
Cooking facilities in bedroom (yes vs. no)	0.42 [0.25–1.08]	0.47 [−0.04–0.99]	0.43 [−0.25–1.11]	0.33 [−0.24–0.91]	0.31 [−0.09–0.71]	0.23 [−0.17–0.62]
Parent’s perception of neighbourhood shop supplies (enough vs. not enough)	0.50 [−0.21– 1.20]	0.19 [−0.39–0.78]	0.50 [−0.29–1.28]	0.39 [−0.33–1.12]	0.34 [−0.16–0.83]	0.28 [−0.19–0.75]
**Parent’s health, behaviours, and social network (model 2) ^2^**						
***Parent’s health***						
Depression symptoms (yes vs. no)	0.25 [−0.72–1.22]		−0.70 [−1.30– −0.09]	−0.32 [−1.01–0.38]	−0.06 [−1.03–0.91]	
BMI (kg/m^2^)	0.09 [0.03–0.16]	0.03 [−0.02–0.08]	0.03 [−0.02–0.08]		0.00 [−0.03–0.03]	
***Parent’s behaviour***						
Sleep time (hours)	0.20 [0.07–0.34]	0.17 [0.08–0.25]	0.09 [−0.07–0.26]		0.08 [−0.06–0.21]	0.10 [−0.01–0.21]
***Parent’s social network***						
Contact with all family members (PCA score)	−0.20 [−0.43–0.02]	0.01 [−0.17–0.18]	−0.01 [−0.22–0.20]		0.15 [−0.02–0.32]	0.13 [−0.03–0.30]
Contact with friends (≥1 vs. never in the last 12 months)	0.88 [0.02–1.73]	0.15 [−0.23–0.53]	0.71 [0.19–1.23]	0.61 [0.13–1.10]	−0.09 [−0.58–0.39]	
**Parent–child interactions (model 3) ^3^**						
Child’s bedtime (hours)	−0.62 [−0.93– −0.31]	−0.05 [−0.35–0.25]	−0.39 [−0.72– −0.06]	−0.23 [−0.57–0.10]	−0.19 [−0.49–0.11]	−0.31 [−0.58– −0.04]
Parent helps with homework (> vs. ≤30 min/day)	0.88 [0.28–1.48]	0.19 [−0.50–0.89]	0.63 [0.09–1.17]	0.49 [−0.01–1.00]	−0.65 [−1.14–−0.16]	−0.41 [−0.83–0.01]
Child meets friends (yes vs. no)	0.67 [−0.51–1.85]		0.12 [−0.38–0.62]		0.62 [0.06–1.19]	0.67 [0.18–1.17]
**Child characteristics (model 4) ^4^**						
Child’s age (years)	−0.03 [−0.32–0.27]		0.03 [−0.11–0.18]		−0.14 [−0.26– −0.02]	−0.08 [−0.19–0.04]

BMI: body mass index; CI: confidence interval; CU: consumption unit; PCA: principal component analysis; SSBs: sugar-sweetened beverages; T: tertile. ^1^ Model 1: adjusted for level 1 variables associated with *p* < 0.20 in unadjusted analyses; ^2^ model 2: adjusted for level 2 variables associated with *p* < 0.20 in unadjusted analyses and level 1 variables in model 1 associated with *p* < 0.20; ^3^ model 3: adjusted for level 3 variables associated with *p* < 0.20 in unadjusted analyses and level 1 and 2 variables in models 1 and 2 associated with *p* < 0.20; ^4^ model 4: adjusted for level 4 variable associated with *p* < 0.20 in unadjusted analyses and level 1, 2, and 3 variables in models 1, 2, and 3 associated with *p* < 0.20.

**Table 4:** Associations (β [95% CI]) between lifestyle patterns and children’s physical and mental health: unadjusted and adjusted models. The ENFAMS survey.

	Girls (n = 114)		Boys (n = 121)			
	Diverse Diet, Sleep		Diverse Diet, Outdoor Play, Sleep		Unbalanced Diet, Outdoor Play, Sleep	
	Unadjusted	Adjusted ^1^	Unadjusted	Adjusted^1^	Unadjusted	Adjusted^1^
***Physical health***						
BMI z-score (WHO references)	0.17 [0.01–0.32]	0.09 [−0.14–0.33]	−0.01 [−0.19–0.16]	0.05 [−0.13–0.24]	0.08 [−0.15–0.31]	0.12 [−0.12–0.37]
Haemoglobin concentration (HemoCue^®^ Hb201+ System)	−0.15 [−0.36–0.06]	−0.11 [−0.27–0.06]	−0.02 [−0.14–0.10]	−0.11 [−0.23–0.01]	0.08 [−0.14–0.30]	0.09 [−0.06–0.24]
***Mental health***						
***Child-reported***						
*Internalizing*						
Specific phobia symptoms (DI score)	0.00 [−0.12–0.11]	0.05 [−0.08–0.19]	−0.16 [−0.32–0.01]	−0.20 [−0.39–−0.01]	0.02 [−0.22–0.26]	−0.01 [−0.19–0.17]
Separation anxiety symptoms (DI score)	0.00 [−0.11–0.12]	0.12 [−0.03–0.27]	−0.10 [−0.24–0.04]	−0.22 [−0.37–−0.06]	0.07 [−0.15–0.30]	−0.01 [−0.16–0.14]
Generalized anxiety symptoms (DI score)	−0.03 [−0.20–0.13]	−0.04 [−0.20–0.13]	−0.10 [−0.28–0.09]	−0.21 [−0.39– −0.04]	−0.10 [−0.32–0.13]	−0.14 [−0.29–0.01]
Depression/dysthymia symptoms (DI score)	−0.06 [−0.18–0.07]	−0.11 [−0.24–0.03]	−0.09 [−0.27–0.09]	−0.14 [−0.33–0.05]	−0.03 [−0.26–0.19]	−0.05 [−0.22–0.11]
*Externalizing*						
Opposition symptoms (DI score)	0.03 [−0.10–0.15]	−0.05 [−0.18–0.08]	−0.04 [−0.23–0.15]	−0.16 [−0.35–0.03]	−0.03 [−0.26–0.20]	0.01 [−0.16–0.17]
Conduct problem symptoms (DI score)	−0.04 [−0.20–0.12]	−0.03 [−0.14–0.08]	−0.03 [−0.20–0.13]	−0.06 [−0.24–0.12]	0.04 [−0.20–0.29]	0.02 [−0.18–0.21]
Hyperactivity–inattention symptoms (DI score)	0.01 [−0.11–0.12]	−0.03 [−0.16–0.09]	−0.05 [−0.24–0.15]	−0.10 [−0.28–0.08]	0.00 [−0.25–0.24]	0.01 [−0.17–0.19]
*Well-being*						
Strength and competencies (DI score) ^2^	−0.09 [−0.22–0.04]	0.00 [−0.13–0.13]	0.08 [−0.10–0.26]	0.09 [−0.11–0.29]	0.01 [−0.29 – 0.32]	−0.26 [−0.53–0.01]
***Parent-reported***						
*Internalizing*						
Emotional symptoms (SDQ score)	−0.01 [−0.14–0.13]	−0.01 [−0.15–0.13]	−0.15 [−0.28– −0.02]	−0.08 [−0.25–0.10]	−0.34 [−0.53–−0.16]	−0.32 [−0.50– −0.14]
Peer relationship problems (SDQ score)	−0.19 [−0.35– −0.03]	−0.24 [−0.40–−0.09]	−0.12 [−0.23– −0.01]	−0.12 [−0.27–0.03]	−0.01 [−0.21–0.20]	−0.03 [−0.20–0.14]
*Externalizing*						
Conduct problem symptoms (SDQ score)	−0.07 [−0.24–0.11]	−0.15 [−0.30–0.01]	−0.02 [−0.14–0.10]	−0.09 [−0.25–0.07]	−0.02 [−0.20–0.16]	−0.03 [−0.23–0.18]
Hyperactivity–inattention symptoms (SDQ score)	−0.09 [−0.26–0.08]	−0.07 [−0.25–0.12]	−0.15 [−0.25– −0.05]	−0.20 [−0.34– −0.06]	0.05 [−0.11–0.21]	0.01 [−0.16–0.18]
*Well-being*						
Prosocial behaviours (SDQ score) ^2^	0.16 [0.04–0.27]	0.31 [0.17–0.45]	−0.01 [−0.17–0.15]	−0.02 [−0.23–0.19]	0.00 [−0.24–0.23]	−0.14 [−0.36–0.09]

BMI: body mass index; CI, confidence interval; DI: Dominic Interactive, SDQ: Strength and Difficulties Questionnaire, WHO: World Health Organization. All outcomes were standardized. ^1^ Adjusted for: household—food insecurity, monthly income, housing insecurity; parent—age, region of birth, education level, employment status, living without partner, administrative status, French proficiency, number of children living with the parent in shelter, depressive symptoms, contact with friends, perceived health status, anaemia, exposure to domestic violence, whether parent helps with homework; child—age, birthweight, reported dislike of the family’s accommodation, reported having been bullied at school, with health problem that requires specific care. ^2^ Higher values are positive.

## Data Availability

The datasets used and/or analysed in the current study are available from the corresponding author on reasonable request.

## Supplementary Materials

The following supporting information can be downloaded at: https://www.mdpi.com/article/10.3390/ijerph192316276/s1, Supplementary Material S1: Family socio-ecological factors; Supplementary Material S2: Missing data; Supplementary Material S3: Sensitivity analyses; Table S1: Factor loadings for the “Contact with all family members” pattern. The ENFAMS survey; Table S2: Proportions of missing data for our study sample (n = 235) and imputation models for variables used in the analyses. The ENFAMS survey; Table S3: Factor loadings for lifestyle patterns in girls and boys aged 6–12 years (derived from the complete case analysis). The ENFAMS survey; Table S4: Factor loadings for lifestyle patterns in girls and boys aged 6–12 years (derived from complete cases and the polychoric matrix). The ENFAMS survey; Table S5: Associations between lifestyle patterns and children’s dichotomized physical and mental health scales: adjusted models. The ENFAMS survey; Table S6: E-values for effect estimates and confidence interval (CI) limits for associations between lifestyle patterns and children’s physical and mental health. The ENFAMS survey.

## Abbreviations

BMI: body mass index; CI, confidence interval; CU, consumption unit; DI, Dominic Interactive; EBRBs, energy balance-related behaviours; LP, lifestyle pattern; PCA, principal component analysis; SD, standard deviation; SEP, socio-economic position; SDQ, Strength and Difficulties Questionnaire; SSBs, sugar-sweetened beverages; WHO, World Health Organization.
