# Supplementary material for: Lifestyle Patterns of Children Experiencing Homelessness: Family Socio-Ecological Correlates and Links with Physical and Mental Health
Source: Int J Environ Res Public Health. 2022 Dec 5;19(23):16276. doi: 10.3390/ijerph192316276 (PMC9737210; doi:10.3390/ijerph192316276)
Supplement: Supplementary file 1 [file ijerph-19-16276-s001.zip › ijerph-2034634-supplementary.pdf]

Supplementary data

Supplementary material 1. Family socio-ecological factors.

Socio-economics, socio-demographics, and living conditions.

- Socio-economic position

Parent's education level: High school diploma or higher vs less than a high school diploma.

Parent's employment status: Employed - works with or without a work contract, works as a volunteer, student or intern - vs unemployed - not working and looking for a job, not working and not looking for a job.

Monthly household income: Household monthly income was calculated as the total household income divided by the number of people in the consumption unit (CU) on the basis of the Organisation for Economic Co-operation and Development (OECD) scale: [1] one CU for the first adult in the household, 0.5 CU for the other persons aged 14 years or older, 0.3 CU for the children under 14 years. It was used as a dichotomous variable (>28 euros/CU vs. ≤28 euros/CU – tertile 1).

- Socio-demographics

Parent's region of birth: Sub-Saharan Africa vs the rest of the world, i.e. Commonwealth of Independent States, North Africa, other African countries, European Union, Asia, France, Near and Middle East, Other Europe, other countries of North and South America, and Oceania, including Australia.

Parent's proficiency in French: Yes vs no – no difficulty among the following in the everyday life: understand, speak, read, and write in French.

Parent's administrative status: regularized- in possession of a temporary or permanent residence permit vs not regularized - in possession of refugee status or a residence permit application receipt or without a valid residence permit/document.

- Living conditions

Parent lives with partner: Yes vs no.

Number of children living with the parent in shelter: Number of children of the respondent who live with the respondent, one vs more.

Household food security: Food insecurity status was assessed using the French version of the Household Food Security Module (HFSSM) questionnaire.[2,3] The HFSSM questionnaire includes a preliminary question and 18 items (10 related to adults and 8 to children). A single score ranging from 0 to 18 was calculated as the total number of affirmative responses, i.e. "yes" or "sometimes/often" to the 18 questions. This score was divided into 2 categories defined by the usual thresholds:[4,5] food secure (score <3) vs not food secure (score≥3).

Housing instability: Computed from time since the family became homeless and the number of moves in the last year, and dichotomized as >2 vs ≤ 2.

Cooking facilities in the bedroom: Yes vs no.

Parent's perception of the shop supplies in the neighbourhood: Initially 4 possible answers to indicate level of satisfaction: totally agree, somewhat agree, somewhat disagree, totally disagree - dichotomized into perceived as enough (totally agree or somewhat agree) vs not enough (somewhat disagree or totally disagree).

Supplementary data

Parent’s health, behaviours, and social network.

- Parent's health status

Body mass index (BMI), continuous (kg/m^2).

Depressive symptoms: Symptoms of depression were assessed by the Composite International Diagnostic Interview (CIDI) questionnaire[6,7] and further dichotomized as yes vs no.

- Parent's health behaviours

Parent's sleep time: Continuous, hours/night.

- Parent's social network

Contact with family members: Score on the first principal component identified by principal component analysis with the following variables: the number of contacts with different family members (including father, mother, someone considered as father/mother, siblings, other family members), not living with the parent-child dyad each month in the preceding 12 months. The semi-quantitative response range was: 1: everyday, 2: several times per week, 3: several times per month, 4: once a month; 5: at least once a year, 6: Never – **Supplementary Table S1**.

Contact with friends: invited for special events in the last 12 months - ≥1 vs. never.

Parent-child interactions.

Child's bed time: Continuous, hours.

Child meets friend: Yes vs no.

Parent helps with homeworks: Time spent per day since the beginning of the school year, i.e. September, to help the child with homework: initially collected as minutes per day and categorized as: ≥ vs. <30 minutes/day.

Child characteristics.

Child's age: Continuous, years.

Supplementary data

Supplementary material 2. Missing data.

In our analysis sample of 235 children, of all the variables studied (exposures, outcomes, or covariates), six had no missing data (parent's proficiency in French, parent's region of birth, number of sheltered children, parent's contact with all family members, parent's age, and child's age). Of the remaining variables, missing data rates ranged from 0.4% for the “parent's education level” to 32.3 % for the “parent lives with partner” variables (**Supplementary Table S2**).

Multiple imputation. Variables in the imputation model included those used in the models of our main analyses (**Supplementary Table S2**). Data were imputed by chained equations with the ‘mice’ R package. Predictive mean matching was used for continuous variables with missing values and logistic regression for binary variables (**Supplementary Table S2**).

We generated 20 independent imputed datasets. Estimates were pooled according to Rubin’s rule.[8]

Supplementary data

Supplementary Table S1. Factor loadings for the *Contact with all family member* pattern. The ENFAMS survey.

	n=235	
	Mean (SD)	Pattern 1
Mother[1]	1.95 (2.18)	0.60
Father[1]	2.25 (1.96)	0.68
Played parents role[1]	1.53 (2.22)	0.29
Siblings[1]	3.20 (1.66)	0.76
Other family members[1]	3.80 (2.20)	0.53
Variance explained (%)		35.8
Label		Contact with all family member

SD: Standard deviation.

[1]Likert scale – 1: everyday, 2: several times per week, 3: several times per month, 4: once a month; 5: at least once a year, 6: Never.

Supplementary data

Supplementary Table S2. Proportions of missing data for our study sample (n=235) and imputation models for variables used in the analyses. The ENFAMS survey.

	Type	Imputation model	% Missing
**Socio-economic, socio-demographic, and living conditions**			
Socio-economic position			
Parent's education level	Dichotomous	Logistic	0.4
Parent's employment status	Dichotomous	Logistic	0.4
Household monthly income	Dichotomous	Logistic	3.4
Socio-demographics			
Parent's proficiency in French	Dichotomous	Logistic	0.0
Parent's region of birth	Dichotomous	Logistic	0.0
Parent's administrative status	Dichotomous	Logistic	3.4
Living conditions			
Parent lives with partner	Dichotomous	Logistic	32.3
Number of sheltered children	Dichotomous	Logistic	0.0
Household food security	Dichotomous	Logistic	5.1
Housing insecurity	Dichotomous	Logistic	3.8
Cooking facilities in the bedroom	Dichotomous	Logistic	1.3
Parent's perception of the shop supplies	Dichotomous	Logistic	4.3
**Parent's health, behaviour, and social network**			
Parent's health			
Depressive symptoms	Dichotomous	Logistic	8.5
BMI	Quantitative	Predictive mean matching	16.2
Parent's behaviour			
Sleep time	Quantitative	Predictive mean matching	2.6
Parent's social network			
Contact with all family members	Quantitative	Predictive mean matching	0.0
Contact with friends	Dichotomous	Logistic	1.7
**Parent-child interactions**			
Child's bedtime	Quantitative	Predictive mean matching	5.5
Parent helps with homeworks	Dichotomous	Logistic	12.3
Child meets friends	Dichotomous	Logistic	3.8
**Child characteristics**			
Child's age	Quantitative	Predictive mean matching	0.0
**Child's health outcomes**			
Physical health			
BMI z-score (WHO references)	Quantitative	Predictive mean matching	11.1
Haemoglobin concentration	Quantitative	Predictive mean matching	11.9
Mental health			
Child-reported (DI scores)			
Specific phobia symptoms	Quantitative	Predictive mean matching	13.2
Separation anxiety symptoms	Quantitative	Predictive mean matching	13.2
Generalized anxiety symptoms	Quantitative	Predictive mean matching	13.2
Depression/dysthymia symptoms	Quantitative	Predictive mean matching	13.2
Opposition symptoms	Quantitative	Predictive mean matching	13.2
Conduct problems	Quantitative	Predictive mean matching	13.2
Hyperactivity-inattention symptoms	Quantitative	Predictive mean matching	13.2
Strength and competencies	Quantitative	Predictive mean matching	13.2
Parent-reported (SDQ scores)			
Emotion symptoms	Quantitative	Predictive mean matching	3.4
Peer relationship problems	Quantitative	Predictive mean matching	3.4
Conduct problems	Quantitative	Predictive mean matching	3.4
Hyperactivity-inattention symptoms	Quantitative	Predictive mean matching	3.4
Prosocial behaviours	Quantitative	Predictive mean matching	3.8
**Other variables**			
Parent's age	Quantitative	Predictive mean matching	0.0
Parent's perception of own health	Dichotomous	Logistic	0.4
Parent's anaemia	Dichotomous	Logistic	17.4
Parent's experience of domestic violence the past 12 months	Dichotomous	Logistic	20.4
Child's birth weight	Quantitative	Predictive mean matching	31.5
Child's report of experience of bullying	Dichotomous	Logistic	14.9

Supplementary data

Child's report of dislike of the family accommodation	Dichotomous	Logistic	10.2
Child with health problem that requires specific care	Dichotomous	Logistic	3.0

BMI, body mass index; DI: Dominic interactive; SDQ: Strength and Difficulties questionnaire; WHO: World Health Organization.

Supplementary data

Supplementary material 3. Sensitivity analyses.

Supplementary Table S3. Factor loadings for lifestyle patterns in girls and boys aged 6-12 years (derived from complete case analysis). The ENFAMS survey.

	Girls (n=98)	Boys (n=91)	
	LP1	LP1	LP2
Yogurts[1]	**0.56**	**0.48**	0.05
Fish[1]	**0.52**	**0.48**	**-0.54**
Fruit[1]	**0.58**	**0.77**	0.02
Vegetables[1]	**0.52**	**0.71**	0.07
Rice/pasta[1]	**0.26**	**0.51**	**-0.35**
Bread[1]	**0.35**	-0.08	**0.72**
SSBs[1]	**0.67**	**0.37**	**0.45**
French fries[1]	**0.39**	**0.48**	0.01
Screen time[2]	-0.06	-0.15	-0.08
Outdoor play[3]	-0.08	**0.26**	**0.53**
Sleep[2]	**0.51**	**0.30**	**0.26**
Variance explained (%)	21.8	21.8	13.8
Label	Diverse diet, sleep	Diverse diet, outdoor play, sleep	Unbalanced diet, outdoor play, sleep

SSBs, sugar-sweetened beverages; LP, lifestyle pattern.

[1]Times/week.
[2]Hours/day.
[3]Days/week.
In bold, factor loadings >0.20 or <-0.20.

Supplementary data

Supplementary Table S4. Factor loadings for lifestyle patterns in girls and boys aged 6-12 years (derived from complete cases and polychoric matrix). The ENFAMS survey.

	Girls (n=98)	Boys (n=91)	
	LP1	LP1	LP2
Yogurts[1]	**0.66**	**0.56**	0.05
Fish[1]	**0.61**	**0.46**	**-0.66**
Fruit[1]	**0.47**	**0.62**	**0.24**
Vegetables[1]	**0.52**	**0.74**	0.11
Rice/pasta[1]	**0.27**	**0.55**	**-0.38**
Bread[1]	**0.37**	-0.11	**0.76**
SSBs[1]	**0.62**	**0.60**	**0.20**
French fries[1]	**0.41**	**0.53**	-0.15
Screen time[2]	-0.18	-0.14	**-0.21**
Outdoor play[3]	**-0.24**	**0.24**	**0.52**
Sleep[4]	**0.50**	**0.44**	**0.35**
Variance explained (%)	23.9	26.7	17.2
Label	Diverse diet, less outdoor play sleep	Diverse diet, outdoor play, sleep	Unbalanced diet, outdoor play, sleep

SSBs, sugar-sweetened beverages; LP, lifestyle pattern.

[1] Four categories: 1. Never; 2. Less often; 3. Several times a week; 4. Every day.
[2] Four categories: 1. Never; 2. Less than 1 hour per day; 3. 1-2 hours per day; 4. More than 2 hours per day.
[3] Four categories: 1. Never; 2. 1 to 3 days a week; 3. 4 to 6 days a week; 4. Every day.
[4] Four categories: 1. <9 hours/day; 2.]9;10[hours/day; 3. 10 hours/day; 4. >10hours/day.
In bold, factor loadings >0.20 or <-0.20.

Supplementary data

Supplementary Table S5. Associations between lifestyle patterns and children's dichotomized physical and mental health scales: adjusted models.[1] The ENFAMS survey.

	Girls (n=114)	Boy (n=121)	
	Diverse diet, sleep	Diverse diet, outdoor play, sleep	Unbalanced diet, outdoor play, sleep
	PR[2] (95% CI)	PR[2] (95% CI)	PR[2] (95% CI)
Physical health			
Overweight (yes vs no) [3]	1.34 [1.01–1.78]	1.08 [0.74–1.56]	1.18 [0.83–1.66]
Moderate to severe anaemia (yes vs no) [4]	1.26 [0.85–1.88]	0.73 [0.56–0.94]	0.95 [0.58–1.58]
Mental health			
Child-reported (DI) [5]			
Internalizing			
Specific phobia symptoms (yes vs no)	1.11 [0.86–1.44]	0.79 [0.61–1.04]	0.86 [0.64–1.15]
Separation anxiety symptoms (yes vs no)	1.06 [0.78–1.43]	0.74 [0.52–1.05]	1.12 [0.76–1.65]
Generalized anxiety symptoms (yes vs no)	0.99 [0.61–1.61]	0.59 [0.44–0.79]	0.69 [0.47–1.03]
Depression/dysthymia symptoms (yes vs no)	0.79 [0.55–1.14]	0.58 [0.40–0.82]	0.85 [0.54–1.32]
Externalizing			
Opposition symptoms (yes vs no)	1.11 [0.52–2.37]	0.82 [0.53–1.25]	0.89 [0.59–1.32]
Conduct problem symptoms (yes vs no)	0.87 [0.53–1.42]	0.77 [0.58–1.03]	1.08 [0.77–1.52]
Hyperactivity-inattention symptoms (yes vs no)	1.11 [0.55–2.23]	1.16 [0.75–1.81]	0.98 [0.61–1.59]
Well-being			
Strength and competencies problems (yes vs no)	1.62 [0.67–3.92]	0.84 [0.41–1.72]	1.39 [0.77–2.51]
Parent-reported (SDQ) [6]			
Internalizing			
Emotional symptoms (yes vs no)	0.98 [0.72–1.34]	1.14 [0.79–1.65]	0.60 [0.47–0.76]
Peer relationship problems (yes vs no)	0.72 [0.54–0.96]	0.65 [0.42–1.00]	0.75 [0.40–1.41]
Externalizing			
Conduct problem symptoms (yes vs no)	1.00 [0.65–1.54]	0.80 [0.57–1.13]	0.93 [0.63–1.38]
Hyperactivity-inattention symptoms (yes vs no)	0.99 [0.62–1.61]	0.56 [0.41–0.77]	1.09 [0.69–1.71]
Well-being			
Prosocial behaviour problems (yes vs. no)	0.65 [0.49–0.86]	0.76 [0.50–1.16]	1.33 [0.88–2.02]

CI, confidence interval; DI, Dominic interactive; PR, prevalence ratio; SDQ, Strength and Difficulties questionnaire; SSBs, sugar-sweetened beverages.

[1]Adjusted for: household - food insecurity, monthly income, and housing insecurity; parent - age, region of birth, education level, employment status, living without partner, administrative status, French proficiency, number of children living with parent, depressive symptoms, contact with friends, perceived health status, anaemia, exposure to domestic violence; parent helps with homework; child - age, birth weight, reported to dislike the family's accommodation, reported to be bullied at school report, has health problem that requires specific care.

[2]We followed Zou's recommendation to use Poisson regression to estimate treatment effects on binary outcomes.[9] In addition, we used the IPTW approach to account for covariates because it has particular advantages when estimating prevalence/odds/risk ratios, when non-convergence of covariate-adjusted outcome regression models occurs frequently (here, potentially due to small sample size).[10]

[3] Applying the WHO references cutoffs (weighted % [95% CI]):[11] 48.6 [34.8; 62.4] in girls, 33.8 [23.2; 44.3] in boys.
[4] Applying the WHO references cutoffs (weighted % [95% CI]):[12] 23.1 [7.9; 38.3] in girls, 25.0 [15.2; 34.8] in boys.

[5] Applying the 'probable problems' cutoffs (weighted % [95% CI]):[13] Specific phobia symptoms: 62.0 [49.2; 74.9], separation anxiety symptoms: 32.2 [20.3; 44.1], generalized anxiety symptoms: 30.0 [20.4; 39.6], depression/dysthymia symptoms: 21.3 [10.3; 32.4], Opposition symptoms: 18.1 [10; 26.3], Conduct problem symptoms: 11.9 [3.1; 20.6], Hyperactivity-inattention symptoms: 7.4 [1.9; 12.8], Strength and competencies problems: 8.5 [2.2; 14.8] in girls; Specific phobia symptoms: 39.1 [27; 51.2], separation anxiety symptoms: 25.1 [14.9; 35.2], generalized anxiety symptoms: 19.4 [9.2; 29.7], depression/dysthymia symptoms: 16.3 [7.3; 25.4], Opposition symptoms: 22.6 [10.3; 34.9], Conduct problem symptoms: 33.3 [20.1; 46.5], Hyperactivity-inattention symptoms: 16.4 [7.9; 24.9], Strength and competencies problems: 16.3 [5.8; 26.9] in boys.

[6] Applying the 'abnormal' cutoffs (weighted % [95% CI]):[14] emotional symptoms: 26.7 [16.7; 36.8], conduct problems: 16.9 [5.2; 28.5], hyperactivity/inattention symptoms: 13.5 [5.2; 21.7], peer relationship problems: 25.2 [13.3; 37], prosocial behaviour problems: 10.3 [1.4; 19.1] in girls; emotional symptoms: 30.3 [20.1; 40.6], conduct problems: 28.7 [18.1; 39.2], hyperactivity/inattention problems: 18.7 [10.4; 27.0], peer relationship problems: 15.5 [6.3; 24.7], prosocial behaviour problems: 19.6 [8.8; 30.4] in boys.

Supplementary data

Supplementary Table S6. *E-values* for effect estimates and confidence interval (CI) limits—for associations between lifestyle patterns and children's physical and mental health. The ENFAMS survey.

	Girls (n=114)		Boy (n=121)			
	Diverse diet, sleep		Diverse diet, outdoor play, sleep		Unbalanced diet, outdoor play, sleep	
	for estimate[1]	for CI[2]	for estimate[1]	for CI[2]	for estimate[1]	for CI[2]
Physical health						
BMI z-score (WHO references)	1.39	1	1.26	1	1.47	1
Haemoglobin concentration (HemoCue® Hb201+ system, Angelholm, Sweden)	1.44	1	1.44	1	1.39	1
Mental health						
<u>Child-reported</u>						
Internalizing						
Specific phobia symptoms (DI score)	1.27	1	1.69	1.08	1.10	1
Separation anxiety symptoms (DI score)	1.47	1	1.74	1.31	1.10	1
Generalized anxiety symptoms (DI score)	1.23	1	1.71	1.21	1.41	1
Depression/dysthymia symptoms (DI score)	1.45	1	1.52	1	1.27	1
Externalizing						
Opposition symptoms (DI score)	1.27	1	1.58	1	1.10	1
Conduct problem symptoms (DI score)	1.20	1	1.29	1	1.15	1
Hyperactivity-inattention symptoms (DI score)	1.20	1	1.41	1	1.10	1
Well-being						
Strength and competencies (DI score)	1.00	1	1.39	1	1.80	1
<u>Parent-reported</u>						
Internalizing						
Emotional symptoms (SDQ score)	1.10	1	1.36	1	2.01	1.54
Peer relationship problems (SDQ score)	1.79	1.39	1.47	1	1.20	1
Externalizing						
Conduct problem symptoms (SDQ score)	1.33	1	1.38	1	1.19	1
Hyperactivity-inattention symptoms (SDQ score)	1.55	1	1.69	1.30	1.41	1
Well-being						
Prosocial behaviours (SDQ score)	1.98	1.62	1.15	1	1.53	1

BMI, body mass index; CI, confidence interval; DI, Dominic interactive; SSBs, sugar-sweetened beverages; SDQ, Strength and Difficulties Questionnaire; WHO: World Health Organization.

[1] The *E-values* for effect estimates are the minimum strength of association on the risk ratio scale that an unmeasured confounder would need to have with both the exposure and the outcome to fully explain away the observed exposure-outcome association, conditional on the measured covariates. For example, an unmeasured confounder would need to be associated with both girls' rather healthy lifestyle pattern and prosocial behaviours by risk ratios of 1.98 each, above and beyond the measured covariates, to fully explain away this observed association.

[2] The *E-values* for the limit of the 95% confidence interval (CI) closest to the null denote the minimum strength of association on the risk ratio scale that an unmeasured confounder would need to have with both the exposure and the outcome to shift the confidence interval to include the null value, conditional on the measured covariates. For example, an unmeasured confounder would need to be associated with both girls' rather healthy lifestyle pattern and prosocial behaviours by 1.62-fold each, above and beyond the measured covariates, to shift the lower limit of the confidence interval for this observed association to include the null value.

Supplementary data

References

1. INSEE Définitions et Méthodes- Unité de Consommation. Available online: https://www.insee.fr/fr/ metadonnees/definition/c1802 (accessed on 28 February 2022).
2. Health Canada Canadian Community Health Survey, Cycle 2.2, Nutrition (2004): A Guide to Accessing and Interpreting the Data. 2006.
3. Radimer, K.L.; Radimer, K.L. Measurement of Household Food Security in the USA and Other Industrialised Countries. *Public Health Nutr.* **2002**, *5*, 859–864, doi:10.1079/PHN2002385.
4. Bickel, G.; Nord, M.; Price, C.; Hamilton, W.; Cook, J. Guide to Measuring Household Food Security: Revised 2000. 2000.
5. National Research Council *Food Insecurity and Hunger in the United States: An Assessment of the Measure*; National Academies Press: Washington, D.C., 2006; p. 11578; ISBN 978-0-309-10132-5.
6. Kessler, R.C.; Üstün, T.B. The World Mental Health (WMH) Survey Initiative Version of the World Health Organization (WHO) Composite International Diagnostic Interview (CIDI). *Int. J. Method. Psychiat. Res.* **2004**, *13*, 93–121, doi:10.1002/mpr.168.
7. Patten, S.B. Performance of the Composite International Diagnostic Interview Short Form for Major Depression in Community and Clinical Samples. *Chronic Dis Can* **1997**, *18*, 109–112.
8. Rubin, D. *Multiple Imputation for Nonresponse in Surveys.*; Wiley.; 1987;
9. Zou, G. A Modified Poisson Regression Approach to Prospective Studies with Binary Data. *American Journal of Epidemiology* **2004**, *159*, 702–706, doi:10.1093/aje/kwh090.
10. Williamson, E.J.; Forbes, A.; White, I.R. Variance Reduction in Randomised Trials by Inverse Probability Weighting Using the Propensity Score. *Statist. Med.* **2014**, *33*, 721–737, doi:10.1002/sim.5991.
11. de Onis, M. Development of a WHO Growth Reference for School-Aged Children and Adolescents. *Bull World Health Organ* **2007**, *85*, 660–667, doi:10.2471/BLT.07.043497.
12. WHO Haemoglobin Concentrations for the Diagnosis of Anaemia and Assessment of Severity Available online: http://www.who.int/vmnis/indicators/haemoglobin/en/.
13. Shojaei, T.; Wazana, A.; Pitrou, I.; Gilbert, F.; Bergeron, L.; Valla, J.-P.; Kovess-Masfety, V. Psychometric Properties of the Dominic Interactive in a Large French Sample. *Can J Psychiatry* **2009**, *54*, 767–776, doi:10.1177/070674370905401107.
14. Shojaei, T.; Wazana, A.; Pitrou, I.; Kovess, V. The Strengths and Difficulties Questionnaire: Validation Study in French School-Aged Children and Cross-Cultural Comparisons. *Soc Psychiat Epidemiol* **2009**, *44*, 740–747, doi:10.1007/s00127-008-0489-8.
